# TNF-α Activating Osteoclasts in Patients with Psoriatic Arthritis Enhances the Recruitment of Osteoclast Precursors: A Plausible Role of WNT5A-MCP-1 in Osteoclast Engagement in Psoriatic Arthritis

**DOI:** 10.3390/ijms23020921

**Published:** 2022-01-15

**Authors:** Shang-Hung Lin, Ji-Chen Ho, Sung-Chou Li, Yu-Wen Cheng, Chung-Yuan Hsu, Wen-Yi Chou, Chang-Chun Hsiao, Chih-Hung Lee

**Affiliations:** 1 Department of Dermatology, Kaohsiung Chang Gung Memorial Hospital and Chang Gung University College of Medicine, Kaohsiung 83301, Taiwan; hongfufu@gmail.com (S.-H.L.); yuwen@cgmh.org.tw (Y.-W.C.); 2 Department of Dermatology, Chiayi Chang Gung Memorial Hospital and Chang Gung University College of Medicine, Chiayi 613, Taiwan; jichenho@cgmh.org.tw; 3 Genomics and Proteomics Core Laboratory, Kaohsiung Chang Gung Memorial Hospital, Chang Gung University College of Medicine, Kaohsiung 83301, Taiwan; raymond.pinus@gmail.com; 4 Division of Rheumatology, Allergy and Immunology, Department of Internal Medicine, Kaohsiung Chang Gung Memorial Hospital and Chang Gung University College of Medicine, Kaohsiung 83301, Taiwan; chungyuango@gmail.com; 5 Department of Orthopaedic Surgery, Kaohsiung Chang Gung Memorial Hospital and Chang Gung University College of Medicine, Kaohsiung 83301, Taiwan; murraychou@yahoo.com.tw; 6 Graduate Institute of Clinical Medical Sciences, College of Medicine, Chang Gung University, Taoyuan 33302, Taiwan; 7 Division of Pulmonary and Critical Care Medicine, Kaohsiung Chang Gung Memorial Hospital and Chang Gung University College of Medicine, Kaohsiung 83301, Taiwan

**Keywords:** psoriatic arthritis, TNF-α, osteoclastogenesis, WNT5A upregulation, MCP-1, osteoclast precursors, anti-TNF-α agents, anti-IL-17 agents

## Abstract

Psoriatic arthritis (PsA) results from joint destruction by osteoclasts. The promising efficacy of TNF-α blockage indicates its important role in osteoclastogenesis of PsA. WNT ligands actively regulate osteoclastogenesis. We investigated how WNT ligands activate osteoclasts amid the TNF-α milieu in PsA. We first profiled the expression of WNT ligands in CD14^+^ monocyte-derived osteoclasts (MDOC) from five PsA patients and five healthy controls (HC) and then validated the candidate WNT ligands in 32 PsA patients and 16 HC. Through RNA interference against WNT ligands in MDOC, we determined the mechanisms by which TNF-α exerts its effects on osteclastogenesis or chemotaxis. WNT5A was selectively upregulated by TNF-α in MDOC from PsA patients. The number of CD68^+^WNT5A^+^ osteoclasts increased in PsA joints. CXCL1, CXCL16, and MCP-1 was selectively increased in supernatants of MDOC from PsA patients. RNA interference against *WNT5A* abolished the increased MCP-1 from MDOC and THP-1-cell-derived osteoclasts. The increased migration of osteoclast precursors (OCP) induced by supernatant from PsA MDOC was abolished by the MCP-1 neutralizing antibody. *WNT5A* and MCP-1 expressions were decreased in MDOC from PsA patients treated by biologics against TNF-α but not IL-17. We conclude that TNF-α recruits OCP by increased MCP-1 production but does not directly activate osteoclastogenesis in PsA.

## 1. Introduction

Psoriatic arthritis (PsA) is a chronic inflammatory joint disease. Unrecognized joint damage can lead to permanent joint deformity and functional impairment [1,2,3]. The joint destruction is associated with multifocal bony erosion and resorption by active osteoclasts, that are derived from precursors of the monocyte/macrophage lineage [4,5,6]. The differentiation of the osteoclasts from their monocyte precursors is tightly regulated by a cascade of integrated signaling steps, including the macrophage colony stimulating factor (M-CSF) and the receptor activator of NF-κB ligand (RANKL) [6]. M-CSF induces the differentiation of osteoclast precursors (OCP) from monocyte precursors, along with the induction of the receptor activator of nuclear factor-κB (RANK) on the cell surface [7]. Enhanced RANKL expression in the synovial lining in PsA triggers the RANK-expressed OCP to differentiate into the multinucleated osteoclasts [8]. In fact, the number of circulatory OCP is increased in PsA. Consistently, tumor necrosis factor-α (TNF-α), one of the major cytokines involved in the pathogenesis of bony resorption in PsA, increases the number of circulating OCP [8]. For the circulatory OCP to migrate into the joints, several chemokines are potentially involved. One major chemokine, the monocyte chemoattractant protein-1 (MCP-1) is found to recruit C-C chemokine receptor type 2 (CCR-2)-expressing circulatory OCP and T cells to local tissue [9,10,11,12]. MCP-1 level is significantly increased in the synovial fluid in PsA patients [13]. 

Besides MCP-1, RANK/RANKL, and M-CSF, wingless-type MMTV integration site family member (Wnt) signaling is important in balancing bone formation and bone resorption by regulating osteoclast differentiation from OCP [14]. Among the 19 Wnt ligands, Wnt3a, Wnt4, Wnt5a, and Wnt16 have been documented to regulate osteoclast differentiation from OCP [14], and Wnt5a also enhances the expression of RANK on OCP [15]. An autocrine loop of Wnt ligands in the osteoclasts and OCP augments the differentiation of OCP into osteoclasts [16,17,18]. In rheumatoid arthritis, an inflammatory arthritis other than PsA, WNT5A was upregulated in the synovium, along with enhanced production of IL-6 and IL-15 [19]. Emerging evidence supports involvement of WNT signaling pathways in the development of PsA, especially the WNT5A-activated signaling cascades [20]. However, the WNT signaling profiles in CD14^+^ monocyte-derived osteoclasts (MDOC) from PsA patients have never been investigated. This study aimed to unravel the regulatory mechanisms involving WNT signaling in osteoclast activation and its interactions with MCP-1 in PsA.

## 2. Results

### 2.1. The Demographics of Patients with PsA and HC

Thirty-two patients with PsA (male/female: 16/16; average age: 48.3 years old) and 16 HC (male/female: 8/8; average age: 46.4 years old) were identified. All the PsA patients had peripheral arthritis, including 31.3% with axial arthritis, 40.6% with dactylitis, and 62.5% with enthesitis (Table 1). 

### 2.2. Increased Transcription and Translation of WNT5A in MDOC and Tissue Osteoclasts from Affected Joints in PsA Patients 

**Figure 1:**
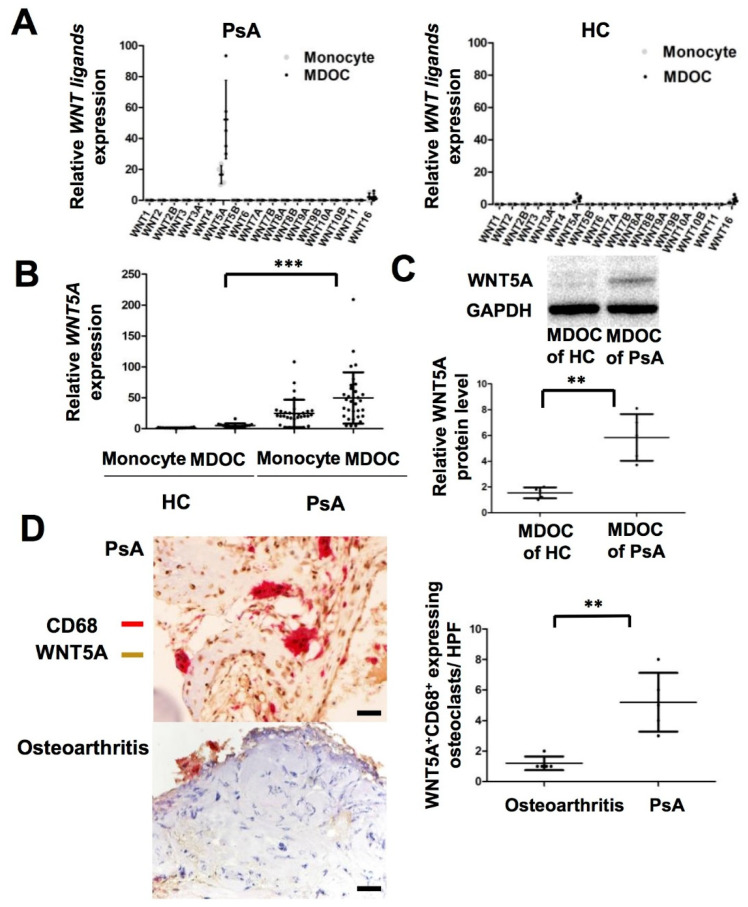
Increased expression of *WNT5A* in CD14^+^ monocyte-derived osteoclasts (MDOC) ex vivo and osteoclasts in destructive joints of PsA. (**A**) RNA samples from CD14^+^ monocytes and MDOC from healthy controls (HC) (*n* = 5) and PsA patients (*n* = 5) were analyzed to profile WNT expression using qRT-PCR. The *WNT5A* was selectively upregulated in MDOC from PsA patients compared to in those from HC. (**B**) The expression of *WNT5A* was measured by qRT-PCR in monocytes and MDOC from HC (*n* = 16) and PsA patients (*n* = 32). (**C**) The protein expression level of WNT5A in MDOC from PsA patients (*n* = 5) and HC (*n* = 5) was measured by Western blotting. The representative blot from MDOC of one HC and one PsA was shown. (**D**) One representative pair of specimens showed marked increased numbers of WNT5A^+^CD68^+^ expressing osteoclasts. Immunohistochemical analyses showed higher numbers of WNT5A (brown)-expressing and CD68 (red)-expressing osteoclasts in the destructive joints of patients with PsA (*n* = 5) than in those from patients with osteoarthritis (*n* = 5). Scale bar: 50 μm. The error bar represents the standard deviation of each data set. ** indicates *p* < 0.01, and *** indicates *p* < 0.001.

We conducted a small pilot study to profile the transcriptional levels of all the *WNT* ligands using qRT-PCR in CD14^+^ monocytes and MDOC from PsA patients (*n* = 5) and HC (*n* = 5). Among the WNT ligands, the expression level of *WNT5A* was selectively increased 13-fold in MDOC from PsA patients compared to the level in MDOC from the HC (*p* < 0.05) (Figure 1A). Although *WNT16* was upregulated in MDOC from HC, the difference did not reach statistical difference. To validate the selective upregulation of *WNT5A* in MDOC from PsA patients, we measured the RNA expression level of *WNT5A* in MDOC from more PsA patients (*n* = 32) and HC (*n* = 16). The results showed that the transcriptional expression of WNT5A was higher in the MDOC from PsA patients than in those from the HC (*p* = 0.0001) (Figure 1B). As we had observed an increase in the expression of *WNT5A* mRNA in the MDOC from PsA patients, we next examined whether the level of the WNT5A protein was similarly increased using Western blotting. The results showed that the level of the WNT5A protein was higher in MDOC from PsA patients (*n* = 5) than in those from HC (*n* = 5) (*p* < 0.01) (Figure 1C). Furthermore, we investigated whether WNT5A expression was increased in osteoclasts in the destructive joints of PsA patients. We collected joint tissues from PsA patients (*n* = 5) and osteoarthritic patients (*n* = 5) who had received joint replacement and stained them with WNT5A and CD68 using immunohistochemical staining. The results showed increased numbers of WNT5A^+^ and CD68^+^ expressing osteoclasts in the joints of the PsA patients compared to those of the osteoarthritis patients (*p* < 0.01) (Figure 1D). We confirmed that the expression of WNT5A protein was selectively increased in the osteoclasts of PsA patients. 

### 2.3. TNF-α Activates WNT5A Pathway, Which Is Independent of Osteoclastogenesis in PsA

**Figure 2:**
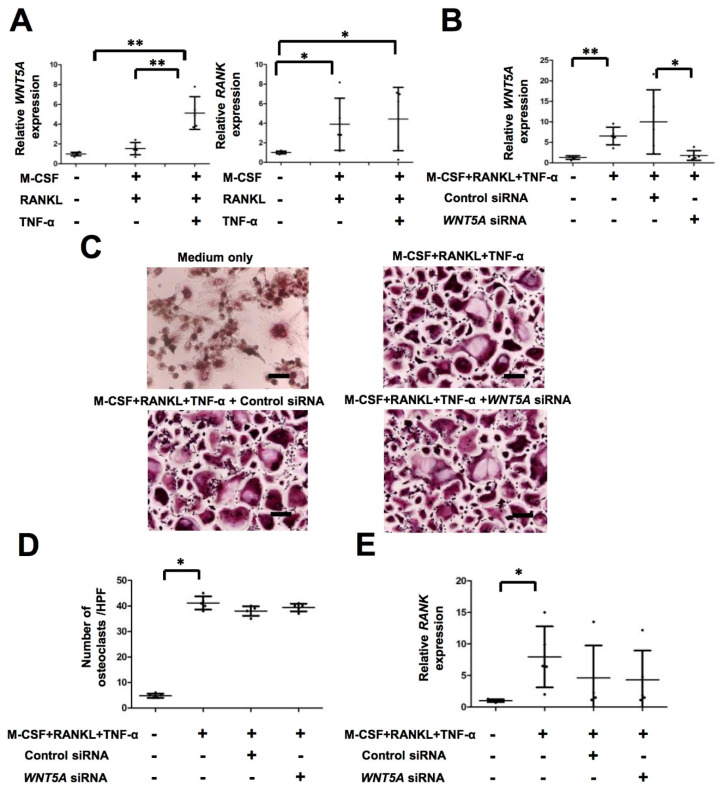
Osteoclastogenesis and *RANK* expression in MDOC is independent of *WNT5A* expression. (**A**) The expression of *WNT5A* and *RANK* in CD14^+^ monocytes after M-CSF and RANKL with/without TNF-α treatment on Day 13 was measured using qRT-PCR (*n* = 5). (**B**) MDOC were transfected with either control siRNA (100 μg/mL) or *WNT5A* siRNA (100 μg/mL) to investigate the dynamic expression level of *WNT5A* during MDOC differentiation using qRT-PCR. (**C**) MDOC transfected with control siRNA or *WNT5A* siRNA were identified morphologically using TRAP staining. The numbers of MDOC were determined (**D**) by averaging the TRAP+ cells in four high-power fields (*n* = 5). Scale bar: 50 μm. (**E**) The relative expression of *RANK* in MDOC transfected with control siRNA or *WNT5A* siRNA was measured using qRT-PCR. The error bar is the standard deviation of each data set. * *p* indicates *p* < 0.05 and ** indicates *p* < 0.01.

WNT5A was highly expressed in the MDOC of the PsA patients. We investigated which cytokines contributed to this increased expression level. RNA samples from monocytes and MDOC were analyzed using qRT-PCR. The results show that the expression of *WNT5A* was significantly increased in MDOC by M-CSF, RANKL and TNF-α treatment compared to that with medium only and/or M-CSF+ RANKL treatment (*p* < 0.05) (Figure 2A). These results indicate that TNF-α significantly increased the expression of *WNT5A* in MDOC from PsA patients. The previous study showed that Wnt5a increased the expression of rank and active osteoclastogenesis in a murine arthritis model [15]. We then investigated whether RANK mediated osteoclastogenesis in PsA. As anticipated, the expression level of *RANK* was higher in MDOC following RANKL and RANKL+ TNF-α treatment than following treatment with medium only (*p* < 0.05) (Figure 2A). We also explored whether *WNT5A* regulated *RANK* or osteoclastogenesis. After *WNT5A* interference, we measured the expression levels of *WNT5A* and *RANK* using qRT-PCR and the number of osteoclasts using TRAP staining. The results showed that *WNT5A* mRNA was significantly downregulated in the *WNT5A* siRNA but not in the control siRNA group (*p* < 0.05) (Figure 2B). The number of TRAP+ osteoclasts was induced after combined M-CSF, RANKL, and TNF-α treatment, and it was not changed by *WNT5A* RNA interference (Figure 2C,D). The increased expression level of *RANK* after combined M-CSF, RANKL, and TNF-α treatment was not inhibited by *WNT5A* siRNA (Figure 2E). These results suggest that *WNT5A* does not directly contribute to active osteoclastogenesis and *RANK* expression in MDOC from PsA patients.

### 2.4. Selective Induction of MCP-1, but Not CXCL1 or CXCL16, by WNT5A in MDOC from PsA Patients

**Figure 3:**
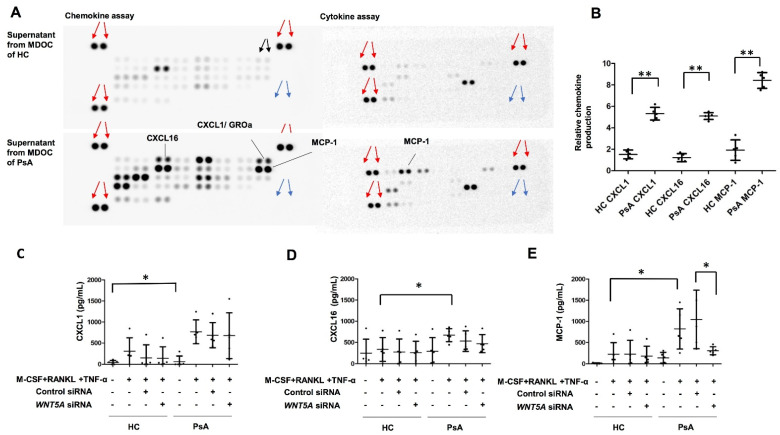
Increased MCP-1 level in supernatants of MDOC from patients with PsA was abrogated by *WNT5A* RNA interference. (**A**) The culture supernatants from MDOC from PsA patients (*n* = 5) and HC (*n* = 5) on Day 13 were assayed using a human cytokine array. Positive controls were marked by red arrows, while the negative controls were marked by blue arrows. The dot marked as black arrows represents the fold change of 1. The average signal intensity of each pair of cytokine spots was quantified using ImageJ and is expressed as the fold change relative to the data for HC. (**B**) CXCL1, CXCL16, and MCP-1 levels in the supernatants from MDOC of PsA patients and of HC were quantified using the data from (**A**) with ImageJ. (**C**–**E**) MDOC from PsA patients (*n* = 5) or HC (*n* = 5) were obtained by incubation with M-CSF, RANKL, and TNF-α, with transfection of control siRNA or WNT5A siRNA. CXCL1 (**C**), CXCL16 (**D**), and MCP-1 (**E**) levels were determined using ELISA in culture supernatants from monocytes or MDOC with RNA interference. The error bar representes the standard deviation of each data set. * indicates *p* < 0.05, and ** indicates *p* < 0.01.

We wanted to determine whether chemokine or cytokine production was increased in the MDOC of the PsA patients compared to those of the HC. The supernatants from the MDOC of the PsA (*n* = 5) patients and HC (*n* = 5) were analyzed using a multiplex chemokine assay. Among 36 chemokines, the results showed higher expression levels of CXCL1, CXCL16, and MCP-1 in the supernatants of the MDOC from the PsA patients than those from the HC (*p* < 0.05) (Figure 3A,B). We further investigated whether WNT5A regulated the production of CXCL1, CXCL16, and MCP-1. The levels of the three cytokines in the supernatants of monocytes and MDOC with/without *WNT5A* RNA interference were measured using ELISA. The results show that the production of CXCL1, CXCL16 and MCP-1, was increased in MDOC from PsA patients and that the enhanced MCP-1 production (but not that of CXCL1 or CXCL16) in the MDOC from the PsA patients was significantly decreased by *WNT5A* blockade (Figure 3C–E). *WNT5A* may regulate the production of MCP-1 in MDOC from PsA patients.

### 2.5. Increased Production of MCP-1 in THP-1-Cell-Derived Osteoclasts by TNF-α Treatment Was Abrogated by WNT5A Interference

**Figure 4:**
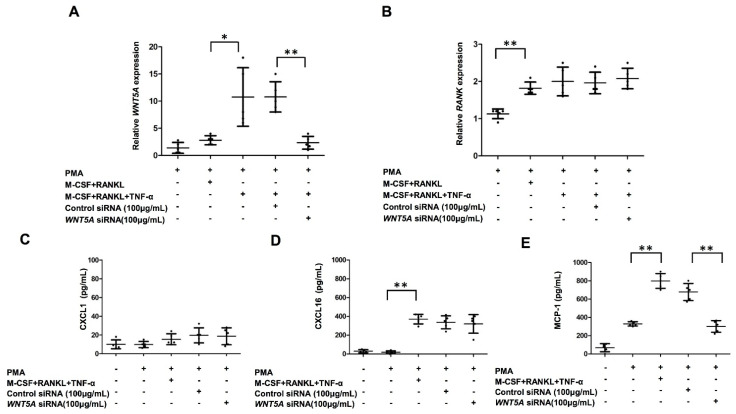
Increased production of MCP-1 in THP-1-cell-derived osteoclasts by combination of TNF-α, M-CSF, and RANKL was abrogated by WNT5A interference. THP-1-derived osteoclasts were transfected with control siRNA or *WNT5A* siRNA at 100 μg/mL on Day 2 for 24 h. On Day 8, the THP-1-derived osteoclasts were obtained for further measurements and experiments. (**A**) The expression of *WNT5A* in THP-1-derived osteoclasts was measured using qRT-PCR. (**B**) The expression of *RANK* in THP-1-derived osteoclasts was measured using qRT-PCR. The levels of CXCL1 (**C**), CXCL16 (**D**), and MCP-1 (**E**) in the supernatant from THP-1-derived osteoclasts transfected with control siRNA or *WNT5A* siRNA were measured using ELISA. Data was obtained from five independent experiments. The error bar is the standard deviation of each data set. * indicates *p* < 0.05, and ** indicates *p* < 0.01.

We found an increased expression of WNT5A in MDOC in patients with PsA. We next investigated whether the upregulation of *WNT5A* by TNF-α could be recapitulated in vitro and sought to decipher its mechanism. We differentiated THP-1 cells into osteoclasts to investigate whether *WNT5A* increased after TNF-α treatment in osteoclasts. RNA samples from THP-1-derived osteoclasts were analyzed using qRT-PCR. The results showed that combined M-CSF, RANKL, and TNF-α treatment induced *WNT5A* expression, which was significantly reduced in cells transfected with *WNT5A* siRNA (100 μg/mL) (Figure 4A). In parallel, we investigated whether *WNT5A* interference abrogated the expression of RANK. The expression level of RANK increased after M-CSF, RANKL and TNF-α treatment, and it was not decreased by *WNT5A* RNA interference. (Figure 4B). We then explored whether *WNT5A* interference abrogated the production of CXCL1, CXCL16, and MCP-1. The concentrations of CXCL1 for THP-1 cells with medium only, THP-1 cells with PMA treatment, THP-1-cell-derived osteoclasts, THP-1-cell-derived osteoclasts with control siRNA, and THP-1-cell-derived osteoclasts with *WNT5A* siRNA (100 μg/mL) were low and did not significantly differ among the groups (Figure 4C). Although the production of CXCL16 was induced after combined M-CSF, RANKL, and TNF-α treatment, it was not changed by *WNT5A* RNA interference (Figure 4D). Notably, the production of MCP-1 was induced after the combined M-CSF, RANKL, and TNF-α treatment but was decreased by more than 50% by *WNT5A* RNA interference (Figure 4E). 

### 2.6. Increased OCP Recruitment Induced by Supernatants of MDOC in PsA Was Abrogated by MCP-1 Blocking

**Figure 5:**
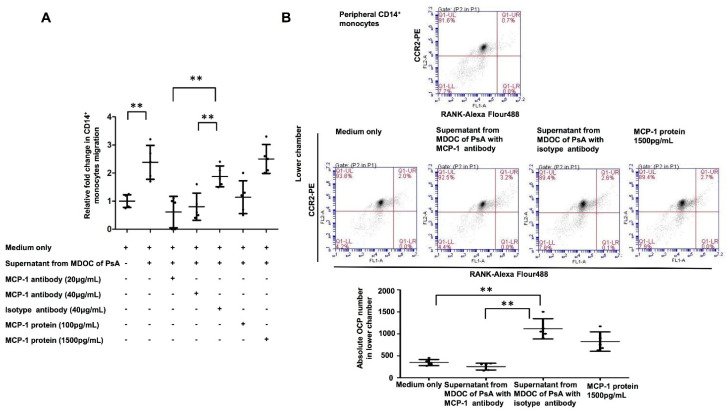
High recruitment of OCP induced by MCP-1 in culture supernatant of MDOC from PsA patients. The supernatants from MDOC of PsA patients (*n* = 5) were collected on Day 13 for Transwell chemotaxis assays. (**A**) Supernatants were mixed with MCP-1 antibody at 20 or 40 ng/mL or corresponding isotype antibody at 40 ng/mL for the lower chamber. In other sets, culture medium supplemented with recombinant MCP-1 protein at 100 and 1500 pg/mL was placed in the lower chamber. The CD14^+^ monocytes from HC (*n* = 5) were placed above the filter (upper chamber) to allow chemotaxis for 1 h. We measured the migration of CD14^+^ monocytes to the lower chamber using a Cell Counting Kit-8. (**B**) The numbers of CD14^+^ monocytes in the lower chamber were determined. OCP, defined as CCR2^+^RANK^+^ expressing CD14^+^ monocytes, were identified by multicolor flow cytometry. The absolute numbers of OCP in the lower chambers were calculated based on the multiplication of the percentage of CCR2^+^RANK^+^ in the Q1 quadrant and the total numbers of cells that migrated into the lower chamber. The secondary antibodies were conjugated with different fluorescent markers (RANK: Alexa Flour488 and CCR2: PE). The isotypes of individual antibodies were used as negative controls. The medium only, supernatant from MDOC of PsA with MCP-1 antibody, supernatant from MDOC of PsA with isotype antibody, or MCP-1 recombinant protein (1500 pg/mL) was added into the lower chamber (*n* = 5 each). The percentages of CCR2^+^RANK^+^ expressing CD14^+^ monocytes in the upper and lower chambers were measured. The error bar represents the standard deviation of each data set. ** *p* indicates < 0.01.

We observed increased MCP-1 in the supernatant of MDOC from PsA patients; we then explored whether this recruited more OCP. The supernatants of MDOC from PsA patients (*n* = 5) were treated with MCP-1 antibody or a mock antibody. We tested the direct effects of supernatant from MDOC with/without MCP-1 antibody on monocyte migration using Transwell assays in which CD14^+^ monocytes from HC were added to the upper chamber and assessed for their response to chemotactic stimuli from the culture supernatant of MDOC from PsA patients, which was added to the lower chamber. The ratio of CD14^+^ monocyte migration was increased when the supernatants were added, and the amount of migration was similar to that induced by the addition of MCP-1 at 1500 pg/mL (Figure 5A). MCP-1 antibody treatment at 20 or 40 μg/mL significantly reduced the enhancement of the migration of MDOC by the supernatant. Furthermore, we wanted to determine whether MCP-1 in the supernatant of MDOC could recruit more CCR2^+^RANK^+^ expressing OCP. The absolute numbers of OCP in the lower chambers were calculated based on the multiplication of the percentage of CCR2^+^RANK^+^ in the Q1 quadrant and the total numbers of cells that migrated into the lower chamber. Coupled with a chemotactic assay and flow cytometry to identify OCP, the data show that the absolute number of OCP in the lower chamber is higher with supernatant from MDOC of PsA with isotype antibody than that with supernatant from MDOC of PsA with MCP-1 antibody (*p* < 0.01) (Figure 5B). The results suggest that increased MCP-1 production in the supernatants of the MDOC from PsA patients recruits high numbers of CCR2^+^RANK^+^ expressing OCP.

### 2.7. Both WNT5A Expression and MCP-1 Production in MDOC of PsA Patients Were Decreased by TNF-α Blockade

**Figure 6:**
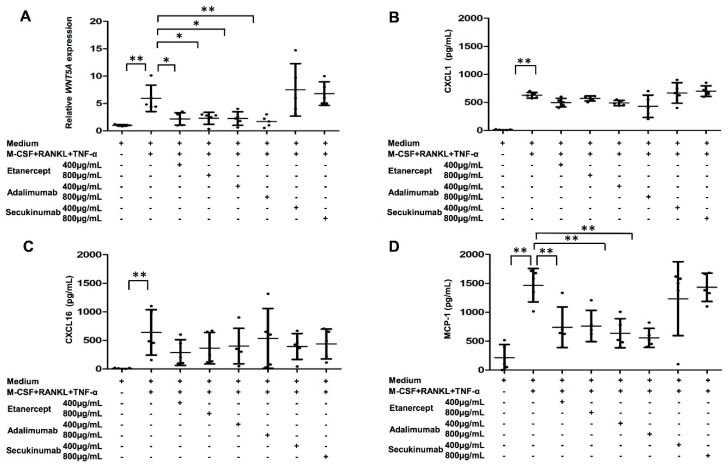
Anti-TNF-α agents inhibit the production of *WNT5A* and MCP-1 in MDOC from PsA patients. MDOC were obtained from 5 PsA patients as described previously. MDOC were treated with anti-TNF-α (adalimumab at 400 and 800 μg/mL or etanercept at 400 and 800 μg/mL) and anti-IL-17A (secukinumab at 400 and 800 μg/mL) agents on Days 3 and 9. On Day 13, the supernatants and RNA samples from MDOC were collected. (**A**) The expression level of WNT5A in MDOC was measured using qRT-PCR. The levels of CXCL1 (**B**), CXCL16 (**C**), and MCP-1 (**D**) in the supernatants were measured via ELISA. The error bar is the standard deviation of each data set. * indicates *p* < 0.05, and ** indicates *p* < 0.01.

We observed that increased MCP-1 production in MDOC recruits OCP in PsA. We next investigated whether TNF-α or IL-17 modulated WNT5A expression in MDOC in PsA. The IL-17 was used as a negative control. For this, MDOC were cultivated from PsA patients as described above. On Days 3 and 9, cells were treated with different concentrations of TNF-α or IL-17 inhibitors. *WNT5A* was upregulated as expected when CD14^+^ cells were treated with combined M-CSF, RANKL, and TNF-α. The upregulation of *WNT5A* was abolished by TNF-α blockers including etanercept and adalimumab, but not the IL-17 blocker secukinumab (Figure 6A). We were also interested in whether the production of MCP-1 from MDOC was mediated by TNF-α or IL-17. The concentrations of CXCL1, CXCL16, and MCP-1 in the supernatants of MDOC treated with or without TNF-α blockers or IL-17 blockers were measured using ELISA. The results show that productions of CXCL1 and CXCL16 increased after M-CSF, RANKL, and TNF-α treatment, they were not changed by anti-TNF-α treatment (etanercept, 400 or 800 μg/mL, or adalimumab, 400 or 800 μg/mL) or anti-IL-17A treatment (secukinumab, 400 and 800 μg/mL) (Figure 6B,C). However, the production of MCP-1 was enhanced in cells that received combined M-CSF, RANKL, and TNF-α treatments. The enhancement was reduced by more than 50% when cells were treated with TNF-α blockers including etanercept and adalimumab, but not with the IL-17A blocker secukinumab (Figure 6D). Overall, we provide compelling evidence that TNF-α mediates MCP-1 production, which may drive the migration of OCP to the PsA joint. 

## 3. Discussion

This is the first study to investigate the role of WNT signaling in MDOC from patients with PsA. Our results reveal higher WNT5A expression levels in MDOC from PsA patients than in those from HC. In addition, WNT5A expression was increased in osteoclasts in the damaged PsA joints compared to that in those from osteoarthritis. MCP-1-mediated OCP migration could be abolished by *WNT5A* RNA interference and blocking TNF-α (but not by blocking IL-17). 

The WNT signaling pathways are involved in physiological bone metabolism [14]. Our results show higher expression levels of *WNT5A* mRNA and protein in MDOC from PsA patients than in those from HC. The increased expression of WNT5A in the osteoclasts of destructive joints confirms its pathogenic role in active osteoclastogenesis in PsA patients. Previous studies showed that TNF-α induced the expression of WNT5A in human monocytes [21] and dental pulp cells [22]. WNT5A expression was increased in the synovial fluids of patients with spondyloarthropathy compared to those with osteoarthritis [23]. Our results consistently show increased expression of WNT5A after TNF-α treatment in the MDOC of PsA patients. Wnt5a promotes osteoclast differentiation and function via RANK expression, thereby enhancing RANKL-induced osteoclastogenesis in mouse models [15]. The knockout of late-stage osteoclast-specific Ror2, a protein downstream of Wnt5a, increases bone mass [24,25]. However, our results show that *WNT5A* interference did not decrease the expression of RANK and inhibit osteoclastogenesis. Instead, WNT5A regulates MCP-1 production, which recruits OCP. In fact, WNT5A has been reported to upregulate MCP-1 expression in macrophages [26]. The WNT5A treatment of human dental pulp cells increased the production of cytokines and chemokines, including IL-8, CXCL1, MCP-1, and CCL5 [22]. This result may reflect the chronic inflammatory burden in PsA.

Increased production of MCP-1, RANTES, CXCL1, CXCL8/IL8, and CXCL9, as well as CCR2 and CCR5, has been described in both the synovial tissue and the synovial fluid of patients with PsA [10,12,27]. Increased MCP-1 in the serum was found to be a potential biomarker for distinguishing PsA from osteoarthritis [28]. Our results show increased concentrations of MCP-1, CXCL1, and CXCL16 in the supernatants of MDOC from PsA patients. Furthermore, the results confirm that *WNT5A* interference could selectively inhibit the production of MCP-1 from MDOC in PsA patients. In addition, the results confirm that the production of MCP-1 by THP-1-cell-derived osteoclasts was regulated by *WNT5A*. Researchers have shown that increased CXCL16 contributed to the retention of CXCR6+ Tc17 cells in PsA synovial fluid [29]. CXCL16 could be produced as a transmembrane-bound chemokine by monocytes, macrophages, and dendritic cells; CXCL16 may contribute to their recruitment and persistence in the inflamed PsA joint [29]. Cytological analysis revealed *CXCL1* mRNA to be located mainly in monocytic cells in the synovial fluid of PsA patients [27]. Hardaway et al. reported that CXCL1 could stimulate osteoclast differentiation in vitro [30].

MCP-1 was induced during TNF-α-mediated osteoclast differentiation [31]. Early work showed that the recruitment of monocytes to the bone surface is mediated by MCP-1 [32,33]. The link between mechanical strain and the onset of arthritis appears to depend on the local recruitment of Ly6high inflammatory monocytes elicited by the mechanostress-induced MCP-1/CCR2 axis [34]. MCP-1 was reported to serve as a chemotactic signal for OCP via the CCR2 receptor [35]. Our results indicate that MCP-1 in the supernatants of MDOC from PsA patients recruits CCR2^+^RANK^+^ expressing OCP. The number of OCP was decreased in PsA patients by successful anti-TNF-α treatment [8]. The good clinical responses to TNF-α inhibition in patients with PsA are well known, although the mechanisms could be multifactorial [36]. One previous study showed that the high numbers of OCP in the peripheral blood of PsA patients were decreased significantly by anti-TNF-α agents [37]. However, the impact of OCP regulation is not known. Anti-TNF-α treatment has been reported to decrease MCP-1 production from cultured mononuclear cells from the synovial fluid of patients with PsA [38]. Our results show that anti-TNF-α agents (etanercept and adalimumab) decreased both *WNT5A* expression and MCP-1 production. Furthermore, the decreased recruitment of OCP through *WNT5A* represents one plausible mechanism independent of blocking osteoclastogenesis for the clinical efficacy of TNF-α inhibitors in treating PsA.

This study has several limitations. First, the case number for the migration study and the regulatory mechanism of MDOC by biologics treatment is low; therefore, further large-scale studies are required for validation. Second, it is difficult to have joint samples from a healthy person. Therefore, we investigated the difference of CD68^+^WNT5A^+^ osteoclasts in the destructive joints of PsA and non-inflammatory arthritis (osteoarthritis). 

In conclusions, this study revealed increased WNT5A expression in MDOC from PsA patients. The increased MCP-1 production from MDOC with TNF-α treatment could be reversed with *WNT5A* RNA interference. Anti-TNF-α agents decreased OCP recruitment through MCP-1 inhibition. 

## 4. Materials and Methods

### 4.1. Isolation of Human Circulatory CD14^+^ Monocytes to Profile WNT Ligands in Patients with PsA and HC 

The clinical manifestations of PsA include peripheral arthritis, axial arthritis, enthesitis or dactylitis, and skin and/or nail involvement. In the PsA group in the present study, all the patients fulfilled the Classification for Psoriatic Arthritis (CASPAR) criteria for the diagnosis of PsA. The cases of PsA were confirmed by both dermatologists and rheumatologists. The HC were examined thoroughly to ensure the absence of any psoriatic lesions or inflammatory joint pain. Patients who had active infections were excluded. All the patients provided written informed consent approved by the IRB of Chang Gung Memorial Hospital (IRB-201802336A3). Patient blood samples were processed to remove red blood cells, followed by the collection of buffy coats enriched with peripheral blood mononuclear cells (PBMCs). Circulating CD14^+^ monocytes were isolated from PBMCs using CD14^+^ MicroBeads (Miltenyi Biotec, Bergisch Gladbach, NRW, Germany). The purity of the CD14^+^ cells after selection was approximately 96.4% according to flow cytometry analysis based on our previous study [39]. Quantitative real-time polymerase chain reaction (qRT-PCR) was performed on the Roche LightCycler^®®^ 96 System (Roche Applied Science, Mannheim, BW, Germany) with the Fast SYBR Green PCR Master Mix (Applied Biosystems; Thermo Fisher Scientific, Inc, Carlsbad, CA, USA) using samples of RNA from the CD14^+^ monocytes of HC and patients with PsA. The PCR program consisted of an initial denaturation at 95 °C for 20 s followed by 45 cycles of qRT-PCR at 95 °C for 3 s (denaturation) and 60 °C for 30 s (annealing, extension, and reading fluorescence). The primer sequences for the different WNT ligands are listed in Table S1. 

### 4.2. Differentiation of Osteoclasts (MDOC) from Human Circulatory CD14^+^ Monocytes 

Purified human CD14^+^ monocytes were seeded at 2.5 × 10^5^ cells/well in 96-well plates containing α-minimum essential medium (α-MEM) with fetal bovine serum (FBS) (10%, *v*/*v*; Invitrogen, Waltham, MA, USA) and M-CSF (20 ng/mL; PeproTech, Rocky Hill, NJ, USA) for 3 days. RANKL (25 ng/mL) and TNF-α (50 ng/mL) (both from PeproTech, Rocky Hill, NJ, USA) were added every 3 days for 9 days to induce osteoclast differentiation. The osteoclasts were identified by staining with tartrate-resistant acid phosphatase (TRAP) on Day 13 using the Acid Phosphate Leukocyte Kit (Sigma, St. Louis, MO, USA). TRAP-stained cells containing three or more nuclei were defined as osteoclasts. [20] The numbers of osteoclasts were counted and averaged from four high-power fields (HPFs) (100×) per well.

### 4.3. Differentiation of Osteoclasts from THP-1 Cells

THP-1 cells (Bioresource Collection and Research Center, Hsinchu, Taiwan) were seeded at 3 × 10^5^ cells/well in 24-well plates containing RPMI 1640 with FBS (10%, *v*/*v*; Invitrogen, Waltham, MA, USA) and PMA (160 nM; Sigma-Aldrich, St Louis, MO, USA) for 1 day. M-CSF (20 ng/mL) was then added on Day 2, and RANKL (25 ng/mL; PeproTech, Rocky Hill, NJ, USA) and TNF-α (25 ng/mL; PeproTech, Rocky Hill, NJ, USA) were added on Days 2 and 5 to induce osteoclast differentiation. The osteoclasts were stained with TRAP on Day 8.

### 4.4. Method for RNA Isolation and cDNA Synthesis

CD14^+^ monocytes, MDOC and THP-1 were lysed in Trizol reagent (ThermoFisher Scientific, Waltham, MA, USA). Total RNA wasextracted using Direct-zol™ RNA Kits (Zymo Research, Irvine, CA, USA) according to the manufacturer’s protocol. We measured the concentration and quality of RNA using Nanodrop spectrophotometer (ThermoFisher Scientific, Waltham, MA, USA). PrimeScript™ RT reagent kit (Takara Biotechnology, Tokyo, Japan) was used to synthesize cDNA according to the manufacturer’s protocol.

### 4.5. Western Blot Analysis for WNT5A in MDOC

A total of 7.5 × 10^5^ MDOC from the patients with PsA and HC were directly lysed in cell lysis buffer (Abcam, Cambridge, UK) containing phosphatase inhibitor cocktail (Thermo Fisher Scientific, Waltham, MA, USA) on ice, then centrifuged at 12,000× *g* at 4 °C for 10 min to collect the supernatants. Protein content was measured using bicinchoninic acid protein assay (BCA Protein Assay kit, ThermoFisher Scientific, Waltham, MA, USA). For electrophoresis, 10 μg protein was loaded to a NuPAGE™ 4 to 12%, Bis-Tris, 1.0 mm, Mini Protein Gel (ThermoFisher Scientific, Waltham, MA, USA). The proteins were then transferred to a PVDF membrane (Merck Millipore, Darmstadt, Germany). Nonspecific binding sites were blocked with 5% BSA/PBS for 1 h at room temperature. The membranes were subsequently incubated overnight at 4 °C with rabbit anti-human WNT5A (1:1000, Invitrogen, Waltham, MA, USA) and mouse anti-human GAPDH (1:1000, Merck Millipore, Darmstadt, Germany) antibodies. After washing to remove unbound primary antibodies, the secondary antibody was added. Anti-rabbit IgG HRP (1:10,000 Rockland, PA, USA) and anti-mouse IgG HRP (1:10,000, eBioscience, Waltham, MA, USA) were used as the secondary antibodies. The band densities were quantified using the Quantity One software (NIH, Bethesda, MD, USA).

### 4.6. Immunohistochemistry (IHC) for WNT5A^+^ and CD68^+^Expressing Osteoclasts in PsA-Affected Joints

Paraffin-embedded slides from destructive PsA and osteoarthritic joints were used for CD68 and WNT5A immunofluorescent staining. The WNT5A/CD68 dual-immunohistochemical procedure was performed using standard reagents and techniques on the Bond-Max Automated Staining System (Leica Biosystems, Wetzlar, Hesse, Germany). The tissue sections were deparaffinized, rehydrated, and boiled to induce epitope retrieval (100 °C for 20 min). They were then incubated with the first primary antibody, rabbit anti-human WNT5A (1:500, Invitrogen, Waltham, MA, USA), followed by analysis using the Bond Polymer Refine detection system (DS9800, Leica Biosystems, Wetzlar, Hesse, Germany) and using DAB as a substrate to produce a brown color. Subsequently, the sections were incubated with the second primary antibody, mouse anti-human CD68 (clone 514H12, 1:800; Leica Biosystems, Wetzlar, Hesse, Germany), followed by analysis on the same detection system using the AP-Red chromogen to generate a red color. Counterstaining with hematoxylin was performed according to the manufacturer’s instructions. The stained sections were examined by light microscopy (Leica Biosystems, Wetzlar, Hesse, Germany), and digital images were captured at 200× magnification. We estimated the number of osteoclasts from 4 randomly selected high power fields (HPF) (200×) per quadrant of the slide. A total of 4 HPFs were chosen from each quadrant.

### 4.7. RNA Interference against WNT5A in MDOC

The CD14^+^ monocytes from patients with PsA and HC were treated with M-CSF (20 ng/mL) for 3 days. The cells were then transfected with either *WNT5A* siRNA or mock siRNA as a negative control (Dharmacon, Lafayette, CO, USA) using Lipofectamine 3000 for 6 h (Invitrogen, Carlsbad, CA, USA). The contents of the si*WNT5A* SMARTpool (Dharmacon, Lafayette, CO, USA) included a mixture of four siRNAs (GUUCAGCUGUCAGAAGUAU, UCAGAUGUCAGAAGUAUAU, GCGACAACAUCGACUAUGG, and GGUCGCUAGGUAUGAAUAA), while the contents of the mock siRNA Control Pools (Dharmacon, Lafayette, CO, USA) included a mixture of four siRNAs (GAAGAACGGAGUAGACUAU, GCACAAGACGACAAGAUAU, CAGAAUGGGUGAUCAUAUC, and GAAUAGGUCAGAUAUGCAA). After transfection, RANKL (25 ng/mL) and TNF-α (50 ng/mL) were added every 3 days for 9 days to induce and maintain osteoclast differentiation. 

### 4.8. Human Cytokine and Chemokine Antibody Array

A Human Cytokine Array Panel A (ARY005; R&D Systems, Minneapolis, MN, USA) and a Human Chemokine Array Kit (ARY017; R&D Systems, Minneapolis, MN, USA) were used to profile 36 cytokines and 31 chemokines, respectively, in the supernatants from cell cultures. The array membranes were incubated in blocking buffer for 1 h at room temperature. Subsequently, 1.5 mL of the sample/antibody mixture was added per well, followed by incubation overnight at 2–8 °C on a rocking platform shaker. The membranes were washed three times in wash buffer (ARY005; R&D Systems, Minneapolis, MN, USA) at room temperature. Next, streptavidin-HRP in array buffer was added, and the membranes were incubated for 30 min at room temperature. The membranes were washed again, followed by the addition of Chemi Reagent Mix (R&D Systems, Minneapolis, MN, USA) for 1 min. The membranes were visualized using a PXi multi-application gel imaging system (Syngene, Cambridge, UK).

### 4.9. Enzyme-Linked Immunosorbent Assay (ELISA) 

MDOC from patients were treated with anti-TNF-α (adalimumab at 400 and 800 μg/mL or etanercept at 400 and 800 μg/mL) and anti-IL-17a (secukinumab at 400 and 800 μg/mL) agents at days 3 and 9. At day 13, we aspired the medium and replaced it with a fresh medium. After 24 h, the supernatants from MDOC were collected for ELISA. THP-1-derived osteoclasts were transfected with control siRNA or WNT5A siRNA at 40 and 100 μg/mL on Day 2 for 24 h. On Day 8, we aspired the medium and replaced it with a fresh medium. After 24 h, the supernatants from THP-1 cell-derived osteoclasts with/without wnt5a siRNA were collected for ELISA. Human cytokine protein levels were measured using ELISA kit (R&D Systems, Minneapolis, MN, USA) for CXCL1, CXCL16, and MCP-1 according to the manufacturer’s protocol. Optical density was determined at 450 nm with VersaMax™ ELISA microplate reader (Molecular Devices, San Jose, CA, USA).

### 4.10. Cell Migration

Cell migration was measured using a ChemoTx^®®^ Disposable Chemotaxis System 96-well plate with polycarbonate filters (8 μm pore size) (Neuro Probe, Gaithersburg, MD, USA). Monocytes (1 × 10^5^ in 25 μL of RPMI 1640 medium/10% FBS) were added to the upper chamber. The lower chamber contained 29 μL of RPMI 1640 medium/10% FBS with or without the MCP-1 chemokine or supernatants from monocytes, and MDOC from patients with PsA. The plates were incubated at 37 °C in 5% CO_2_ for 1 h, and the cells that had migrated into the lower chamber were counted using a Cell Counting Kit-8 (Sigma-Aldrich, St. Louis, MO, USA).

### 4.11. Flow Cytometry for RANK in OCP

We measured the surface expression levels of RANK and CCR-2 on OCP using flow cytometry. OCP were stained with anti-human RANK antibody (1:50, R&D Systems, Minneapolis, MN, USA) or mouse IgG1 isotype antibody (1:50, BioLegend, San Diego, CA, USA) for 20 min at room temperature, followed by incubation with the secondary antibody Goat Alexa Fluor 488-conjugated anti-mouse IgG (H + L) (1:500, ThermoFisher Scientific, Waltham, MA, USA) for 20 min at room temperature. The mouse anti-human CCR2 PE antibodies (1:100, BioLegend, San Diego, CA, USA) or mouse IgG2a, κ PE isotype antibody (1:100, BioLegend, San Diego, CA, USA) was used to identify the CCR-2 receptor. The 7-Aminoactinomycin D (7-AAD) (1:100, Cayman, Ann Arbor, Michigan, USA) was used to identify the non-viable cells. The percentage of osteoclast precursor and the non-viable cell in this migration study was analyzed by multi-color flow cytometry (Figure S1).

### 4.12. Statistical Analyses

An unpaired *t*-test was used to compare mRNA expression of *WNT5A* among PsA patients and HC. In the cases where case numbers of protein expression, WNT ligand expression according to IHC, RANK expression in the osteoclasts, numbers of TRAP+ osteoclasts, numbers of migrating monocytes, and chemokine/cytokine production from osteoclasts were few, we used a Mann–Whitney U Test. All the data are presented as the mean +/− SD. A *p* value < 0.05 was considered statistically significant.

## Figures and Tables

**Table 1:** Demographics of patients with psoriatic arthritis (PsA) and healthy controls (HC).

	Patients with PsA *(n* = 32)	HC (*n* = 16)
Age (years)	48.3 ± 11.0	46.4 ± 12.5
Female sex (no. (%))	16 (50%)	8 (50%)
Weight (kg)	71.6 ± 11.0	69.3 ± 13.6
Psoriasis (years)	15.3 ± 9.3	
Psoriatic arthritis (years)	9.3 ± 7.9	
Previous drug use		
Anti-TNF drugs, anti-IL-12/23, or anti-IL-17 (no. (%))	4 (10)	
Use of methotrexate (no. (%))	21/32 (65.6)	
Use of leflunomide, (no. (%))	10 (25)	
Use of NSAID, (no. (%))	38 (95)	
Patients with specific disease characteristics		
PASI	12.9 ± 8.8	
Peripheral arthritis, (no. (%))	32 (100)	
Peripheral and axil arthritis, (no. (%))	10 (31.3)	
Dactylitis, (no. (%))	13/32 (40.6)	
Enthesitis, (no. (%))	20/32 (62.5)	
Tender-joint count (of 78 joints)	9.9 ± 7.6	
Swollen-joint count (of 76 joints)	6.0 ± 6.7	
Uveitis, (no. (%))	2 (6.3)	

## Data Availability

All data generated or analyzed during this study are included in this published article.

## Supplementary Materials

The following supporting information can be downloaded at: https://www.mdpi.com/article/10.3390/ijms23020921/s1.
