# Supplementary material for: TNF-α Activating Osteoclasts in Patients with Psoriatic Arthritis Enhances the Recruitment of Osteoclast Precursors: A Plausible Role of WNT5A-MCP-1 in Osteoclast Engagement in Psoriatic Arthritis
Source: Int J Mol Sci. 2022 Jan 15;23(2):921. doi: 10.3390/ijms23020921 (PMC8778693; doi:10.3390/ijms23020921)
Supplement: Supplementary file 1 [file ijms-23-00921-s001.zip › ijms-1485630-supplementary.pdf]

Table S1. The primer sequences used in qRT-PCR.

Oligo Name	Oligo Seq
hu-WNT1(F)	TCTTCGGCAAGATCGTCAAC
hu-WNT1(R)	AGTCACACGTGCAGGATTC
hu-WNT2(F)	AACAGAGCTGGCAGGAAG
hu-WNT2(R)	AGAGATAATCGCCCGTTTTCC
hu-WNT2B(F)	CGAGAGGCAGCTTTTGTATATG
hu-WNT2B(R)	CCAGTCAAAGTCCCCACG
hu-WNT3(F)	GTGTTAGTGTCCAGGGAGTTC
hu-WNT3(R)	CATTTGAGGTGCATGTGGTC
hu-WNT3A(F)	ATCAAGATTGGCATCCAGGAG
hu-WNT3A(R)	CAATGGCGTGGACAAAGG
hu-WNT4(F)	GTGCCAGTACCAGTTCCG
hu-WNT4(R)	CACACCTGCCGAAGAGATG
hu WNT5A(F)	CCGGTACTAGCTAACTCCAA
hu WNT5A(R)	CACCATTCCACAGAGAGAGA
hu-WNT5B(F)	AAGGAGTTTGTGGATGCCC
hu-WNT5B(R)	GCTACGTCTGCCATCTTATACAC
hu-WNT6(F)	GAGAGTGCCAGTTCCAGTTC

hu-WNT6(R)	TGATGGCGAACACGAAGG
hu-WNT7A(F)	AAGGTCTTTGTGGATGCCC
hu-WNT7A(R)	GCACTTACATTCCAGCTTCATG
hu-WNT7B(F)	GGATCATGCACAGAAACTTTCG
hu-WNT7B(R)	GCTAGGCCAGGAATCTTGTT
hu-WNT8A(F)	CGAAAACTGTGGCTGTGATG
hu-WNT8A(R)	CTTCCCCTTCTCCAAACTGTC
hu-WNT8B(F)	GTACACCCTGACTAGAAACTGC
hu-WNT8B(R)	CAAACTGCTTGGAAATCGCC
hu-WNT9A(F)	CAGCAAGTTCGTCAAGGAATTC
hu-WNT9A(R)	TGCATGAGCCTGACACG
hu-WNT9B(F)	AGTGCCAGTTTCAGTTCCG
hu-WNT9B(R)	GGAAAGCTGTCTCTTTGAAGC
hu-WNT10A(F)	CTCCTGTTCTTCCTACTGCTG
hu-WNT10A(R)	CACTGTGTTGGCATTGAGC
hu-WNT10B(F)	TCTCGGGATTTCTTGGATTCC
hu-WNT10B(R)	CATTTCCGCTTCAGGTTTTCAG
hu-WNT11(F)	CCAAGCCAATAAACTGATGCG
hu-WNT11(R)	GCACTTACACTTCATTTCCAGAG

hu-WNT6(F)	CAGAAAGATGGAAAGGCACC
hu-WNT16(R)	ATCATGCAGTTCCATCTCTC
hu-RANK(F)	CCATCATCTTTGGCGTTTG
hu-RANK(R)	AGCTGTGAGTGCTTTCCCT

Figure S1. The percentage of osteoclast precursor and the non-viable cell in this migration study was analyzed by multi-color flow cytometry. $CD14^+$ magnetic microbeads were used to identify peripheral monocytes. $1X10^5$ $CD14^+$ monocytes were added to the upper chamber. The $CD14^+$ monocytes migrated into the lower chamber for one hour. Osteoclast precursor was characterized using RANK and CCR2. The secondary antibodies were conjugated with different fluorescence (RANK-Alexa Flour488 and CCR2-PE-A). The isotypes of individual antibodies were used as negative controls. The gating line is set according the corresponding isotype antibody. (B) To determine the percentage of non-viable cell in the migration study. The peripheral $CD14^+$ monocytes, $CD14^+$ monocytes in upper chamber and $CD14^+$ monocytes in lower chamber after migration study, 7-Aminoactinomycin D (Cayman, Ann Arbor, Michigan, US) was used to detect non-viable cells.

(A)

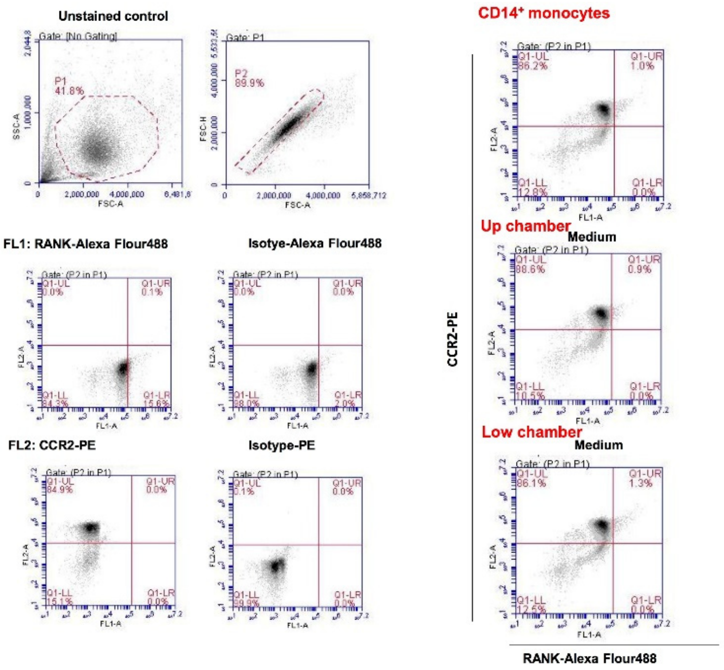

Unstained control
CD14+ monocytes
Up chamber
Medium
Low chamber
Medium
FL1: RANK-Alexa Flour488
Isotye-Alexa Flour488
FL2: CCR2-PE
Isotype-PE
CCR2-PE
RANK-Alexa Flour488

(B)

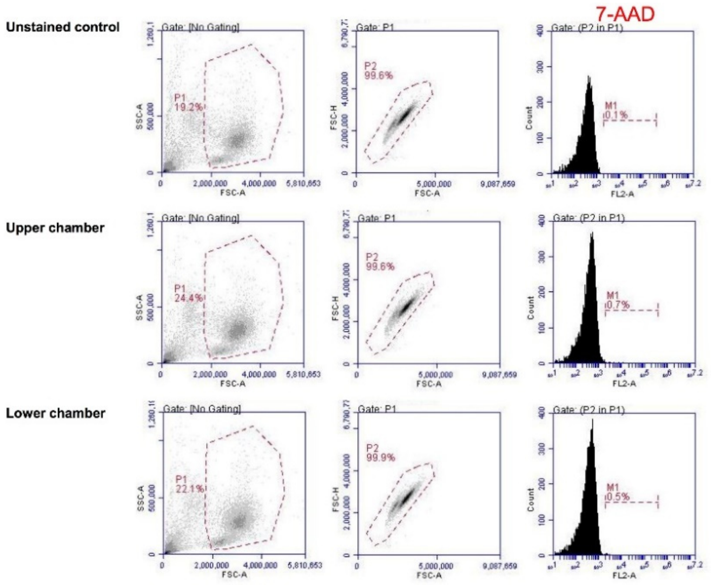

Unstained control
Upper chamber
Lower chamber
7-AAD
